# Outcomes in Patients With Post-Myocardial Infarction Ventricular Tachycardia Ablations: Single vs MultiVessel Infarction

**DOI:** 10.1016/j.jacadv.2026.102644

**Published:** 2026-03-11

**Authors:** Kenji Hashimoto, Samual Turnbull, Max Bickley, Kaimin Huang, Kasun De Silva, Ashwin Bhaskaran, Saurabh Kumar

**Affiliations:** Department of Cardiology, Westmead Hospital, Westmead Applied Research Centre, University of Sydney, Westmead, Australia

**Keywords:** catheter ablation, ischemic cardiomyopathy, ventricular tachycardia

**What is the clinical question being addressed?**

Are outcomes after ventricular tachycardia ablation different in patients with multivessel versus single-vessel infarction?

**What is the Main Finding?**

Multivessel infarction patients had greater scar burden and were associated with worse short- and long-term outcomes, including higher in-hospital mortality and lower ventricular tachycardia–free survival, after ventricular tachycardia ablation.

Catheter ablation (CA) is a well-established treatment to prevent ventricular tachycardia (VT) recurrence in post-myocardial infarction (MI) patients. The location of MI, as well as infarct size, influences the complexity of VT substrate and outcomes after CA.^1^ However, outcomes after VT ablation in patients with multivessel infarction remain undescribed.

From 2018 to 2023, 116 consecutive patients with post-MI who underwent VT ablation were included in our single-center retrospective analysis and divided into 2 groups: single-vessel infarction (n = 82) or multivessel infarction (n = 34). Multivessel infarctions were defined as having evidence of (1) multivessel coronary artery disease on coronary angiography and (2) infarction extended beyond a single coronary artery-dominated area on imaging (transthoracic echocardiography, cardiac computed tomography, or magnetic resonance imaging) and/or electroanatomic mapping. The demographics, procedural data, and long-term outcomes were compared. The primary outcomes were cardiovascular mortality after discharge and VT recurrence after multiple procedures. The safety outcomes are complications, including in-hospital mortality. Continuous variables were compared using t-tests or Mann-Whitney U tests, and categorical variables using the Fisher exact test. Event-free survival was estimated by Kaplan-Meier, compared using log-rank tests. HRs and 95% CIs were calculated using Cox models. For VT recurrence, piecewise HRs (0-30 days and >30 days) were calculated because the hazards were nonproportional. A multivariable Cox model was created for the primary outcomes, including age, gender, left ventricular ejection fraction (LVEF), VT storm, the presence of unrevascularized chronic total occlusions (CTOs), and multivessel infarction. Minor missing data (<5%) were not imputed. The local ethics Committee approved the study.

Our approach to scar-related VT ablation has been previously described.^2^ Briefly, high-density substrate mapping was performed based on the characteristics of induced or spontaneous VT. Radiofrequency ablation was performed using a 3.5-mm tip irrigated catheter. Ablation targets were identified using substrate, pace, activation, and entrainment mapping assisted by a 3-dimensional electroanatomical mapping system.

The multivessel group had a lower LVEF (27% ± 9% vs 37% ± 12%, *P* < 0.01) and more prior coronary artery bypass grafting (71% vs 38%, *P* < 0.01). Age (70 ± 10 vs 69 ± 10 years, *P* = 0.44), male (91% vs 95%, *P* = 0.42), and comorbidities, including atrial fibrillation and diabetes, were not different between the multivessel vs single-vessel groups. The distributions of infarcted vessels are shown in Figure 1. In the single-vessel group, 73% had revascularization of infarcted vessels; in the multivessel group, 53% had revascularization of all infarcted vessels, whereas 47% had at least 1 CTO remaining. Active ischemia was routinely assessed and revascularized before the procedure. The multivessel group showed a trend toward more inducible VTs (3.6 ± 2.1 vs 2.8 ± 2.0, *P* = 0.08), and had lower postablation VT noninducibilities (38% vs 61%; *P* = 0.04). Scar analysis on the left ventricular substrate map was performed in 26 (87%) multivessel and 68 (88%) single-vessel patients. Bipolar (<1.5 mV) and unipolar (<8.3 mV) scar were larger in the multivessel group (96 ± 38 vs 54 ± 34 cm^2^, *P* < 0.01; 141 ± 46 vs 81 ± 47 cm^2^, *P* < 0.01). Epicardial ablation was performed in 7% of single-vessel and 6% of multivessel patients (*P* = 1.00). The multivessel group had a higher complication rate (24% vs 6%, *P* = 0.02); in-hospital mortality comprised 6 patients (18%) in the multivessel group, whereas none in the single-vessel group (*P* < 0.01). Of these 6 patients with in-hospital mortality, 5 presented with VT storm before procedure, and death was attributed to intractable VT in 4 cases and to heart failure and lower limb ischemia due to extracorporeal membrane oxygenation in 1 case each.

**Figure 1:**
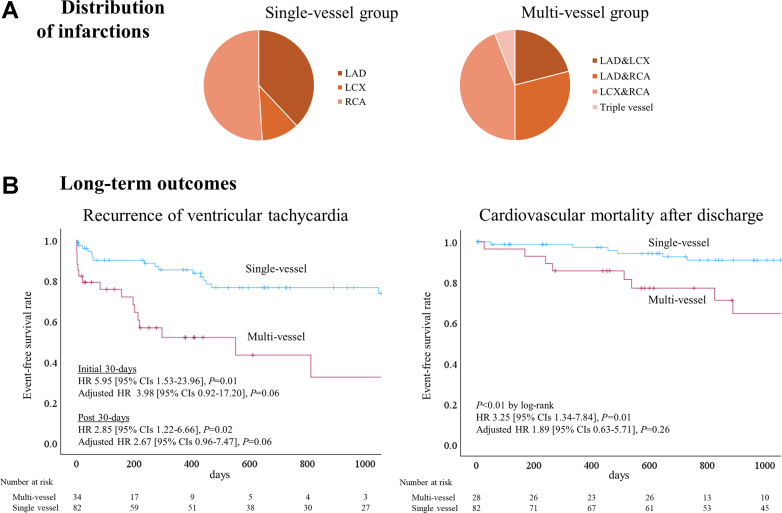
**Distribution of Infarction and Long-Term Outcomes** (A) Distributions of infarctions. (B) Freedom from ventricular tachycardia and cardiovascular mortality. LAD = left anterior descending artery; LCX = left circumflex artery; RCA = right coronary artery.

The median (interquartile range) follow-up was 754 (242-1374) days. The multivessel group had a lower cardiovascular mortality-free survival after discharge in the univariable Cox model but not in the multivariable Cox model (Figure 1). The predominant cause of death was heart failure (76%), followed by intractable VT (14%). The multivessel group had a higher VT recurrence in the initial 30-days and post-30 days in the univariable Cox model; in the multivariable Cox model, the multivessel group showed a trend toward higher VT recurrence in the initial 30-days and post 30-days (Figure 1). In the multivariate Cox model, the presence of unrevascularized CTO was associated with cardiovascular mortality after discharge (adjusted HR: 4.25; 95% CI: 1.56-11.6; *P* < 0.01), but not with VT recurrence (adjusted HR: 0.81; 95% CI: 0.35-1.85; *P* = 0.61). Similarly, LVEF was associated with cardiovascular mortality (adjusted HR: 0.75; 95% CIs: 0.59-0.95 for 5% LVEF increase; *P* = 0.02), but not with VT recurrence (adjusted HR: 0.85; 95% CIs: 0.70-1.03 for 5% LVEF increase; *P* = 0.10).

Samuel et al conducted a Ventricular Tachycardia Ablation versus Escalated Antiarrhythmic Drug Therapy in Ischemic Heart Disease (VANISH) trial substudy and found that the efficacy of VT ablation vs escalated antiarrhythmic therapy varied by MI location. Inferior infarction was associated with lower efficacy of CA compared to noninferior infarction. Notably, the inferior group included 17% with anterior and 16% with posterolateral infarctions, suggesting the presence of multiple infarctions in some patients.^1^ In contrast, another study found similar VT recurrence rates between anterior and inferior infarctions after excluding multivessel infarction cases.^3^ These findings suggest worse outcomes may be linked to multivessel infarction, yet no prior study has specifically examined this group. Our findings suggest that patients with multivessel MI had worse clinical outcomes after VT ablation, possibly due to greater scar burden and complexity of scar distribution. However, the impact of the infarcted territory was not assessed due to the limited sample size in the multivessel group. Further mechanistic studies are warranted to validate our findings.

## Funding support and author disclosures

Dr Kumar is supported by the NSW Early mid-Career Fellowship; he has received speakers’ honoraria from Abbott Medical, 10.13039/501100005035Biotronik, 10.13039/100004374Medtronic, 10.13039/100008497Boston Scientific, Sanofi Aventis, and Biosense Webster Inc; and he has received research funding from Abbott Medical and 10.13039/501100005035Biotronik. Turnbull is supported by a Cardiac Society of Australia and New Zealand Post Graduate Research Scholarship. All other authors have reported that they have no relationships relevant to the contents of this paper to disclose.
